# FEBS fellowships: supporting excellent science for over four decades

**DOI:** 10.1002/2211-5463.13659

**Published:** 2023-07-03

**Authors:** Duncan E. Wright, Alain Krol

**Affiliations:** ^1^ FEBS Open Bio Editorial Office Cambridge UK; ^2^ Architecture et Réactivité de l'ARN, UPR9002, Université de Strasbourg, CNRS Strasbourg France

## Abstract

The Federation of European Biochemical Societies (FEBS) awarded FEBS Long‐Term Fellowships from 1979 until 2020, at which time the scheme was replaced with the FEBS Excellence Award. Over four decades, FEBS awarded a huge number of Long‐Term Fellowships, helping to support and promote the careers of excellent young researchers across Europe. To celebrate the exciting work performed by the FEBS Long‐Term Fellows, we present here a special ‘In the Limelight’ issue of *FEBS Open Bio*, containing four Mini‐reviews and four Research Protocols authored by the fellows themselves. The four Review articles provide timely updates on the respective research fields, while the Research Protocols describe how to perform challenging experimental methods in detail. We hope this issue will be a valuable resource for the community, and a celebration of the high‐quality work done by young scientists.

## Graphical abstract

Over four decades, FEBS awarded a huge number of Long‐Term Fellowships, helping to support and promote the careers of excellent young researchers across Europe. To celebrate the exciting work performed by the FEBS Long‐Term Fellows, we present here a special “In the Limelight” issue of *FEBS Open Bio*, containing four Mini‐reviews and four Research Protocols authored by the fellows themselves. (Photo: Alain Krol.)

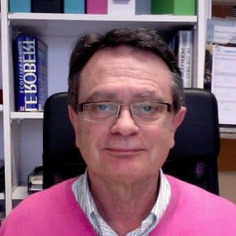

The Federation of European Biochemical Societies (FEBS) was established in 1964 to facilitate connections between biochemists across Europe and soon diversified to support scientists through a number of different programmes, including the introduction of FEBS Long‐Term Fellowships in 1979. These fellowships supported visits by young researchers to laboratories for the purposes of collaboration or training and were originally granted for 1 year, with the possibility of renewal up to a maximum of 3 years. I (Alain Krol) have been fortunate to serve as Chair of the Fellowships Committee of FEBS since 2017, and it has been my pleasure to coordinate the fellowship schemes and support the fellows. As a tradition, Long‐Term Fellows meet every other year during the Fellows Meeting to present their data and share part of their time with the PhD students attending the Young Scientist's Forum. In 2022, a number of former and current FEBS fellows met in Vimeiro (north of Lisbon), the location of the YSF, ahead of the 46th FEBS Congress. In 2021, the Long‐Term Fellowships programme was retired and replaced with a new FEBS Excellence Award scheme, which provides funding for consumables and equipment to an early‐career researcher for 3 years. To mark the retirement of the Long‐Term Fellowships, I am very pleased to introduce this special ‘In the Limelight’ issue of *FEBS Open Bio*, which contains multiple articles authored by FEBS fellows representing one of the last cohorts of the Long‐Term Fellowships programme.

Eight FEBS fellows have contributed either Mini‐reviews or Research Protocols centred around their own research to this issue, resulting in a fascinating cross‐section of recent advances across the molecular biosciences. Research Protocols, which describe an experimental procedure in more detail than a standard article methods section, are a relatively new venture for the journal, with the first articles being published at the end of last year. I am confident that the publication of such protocols, which include a detailed list of reagents and equipment, a step‐by‐step protocol, and a troubleshooting guide, will be of considerable use to young researchers working in related fields.

The Mini‐reviews are all timely and excellent analyses of the state‐of‐the‐art and current knowledge in their respective fields. The first Mini‐review in the issue, authored by the FEBS fellow Matti Turtola together with Juan B. Rodríguez‐Molina, describes the complex series of steps and symphony of proteins required for polyadenylation of mRNA [1]. Recent advances, such as the introduction of AlphaFold, have provided greater insight into this long‐studied phenomenon. The second Mini‐review takes us from the molecular to the cellular level, as FEBS fellow Verónica Miguel together with Rafael Kramann describes metabolic reprogramming in renal fibrosis, a field where omics technology, single cell/nucleus RNA sequencing, and advances in software are facilitating the capture of metabolic profiles at unparalleled spatial and temporal resolution [2]. The third article, by FEBS fellow Stefano L. Giandomenico together with Erin M. Schuman, provides a broad overview of the technologies available to study gene function in mammals, including the use of CRISPR and targeted protein degradation [3]. This article provides a fantastic introduction for those new to the field, and is a useful central hub for obtaining full details on this wide range of methodologies. The fourth and final Mini‐review, by FEBS fellow Yan Ma together with Jade Nicolet, focusses on the intriguing ‘hourglass ‘problem of signalling specificity, whereby different inputs elicit different outputs while using the same signalling intermediates, with this phenomenon being especially pronounced in plants [4].

The issue is rounded out by four excellent Research Protocols, which provide detailed information on the specific use of various experimental techniques: computational analysis of cryo‐EM data, fluorescence microscopy, flow cytometry, and bioinformatic analysis of RNA sequencing data. In the first, FEBS fellow Z. Faidon Brotzakis provides clear and detailed instructions on how to set up and analyse a Metadynamics electron microscopy metaInference (MEMMI) protocol, which combines cryo‐electron microscopy electron density maps with metadynamic‐enhanced‐sampling molecular dynamics to investigate the conformational heterogeneity of protein structures [5]. The second Research Protocol, by FEBS fellow Pierre Santucci and colleagues, describes how to use fluorescence microscopy‐based approaches to study the responses of *Mycobacterium tuberculosis* to acidic microenvironments within human macrophages, which will aid investigation into the pathogenesis of tuberculosis [6]. In the third article, FEBS fellow Geula Hanin together with Anne C. Ferguson‐Smith, describe the use of flow cytometry to enable the isolation and subsequent analysis of mammary adipocytes [7]. Finally, FEBS fellow Valentina Sica and colleagues provide comprehensive details on how to isolate muscle stem cells, extract RNA for sequencing, and analyse the sequencing data [8]. The diversity of topics is testament to the wide range of research supported through the FEBS Long‐Term Fellowship programme.

I am sure this collection will be of wide interest to the readership of *FEBS Open Bio*, and I wish all of the FEBS fellows continued success in their careers and future endeavours. I am sure the new FEBS Excellence Awards will continue to support scientists for many generations to come.

## Conflict of interest

The authors declare no conflict of interest.

## Author contributions

AK and DEW wrote the editorial.
